# Identification of immune-related genes and molecular subtypes associated with preeclampsia via bioinformatics analysis and experimental validation

**DOI:** 10.1186/s41065-025-00458-9

**Published:** 2025-05-29

**Authors:** Tingting Zhao, Ying Peng

**Affiliations:** Department of Obstetrics and Gynecology, The First Affiliated Hospital of USTC, Division of Life Sciences and Medicine, University of Science and Technology of China, Hefei, Anhui China

**Keywords:** PE, Differentially expressed genes, Immune mechanism, Bioinformatics analysis, PCR

## Abstract

**Background:**

Preeclampsia (PE) is a pregnancy disorder that occurs after 20 weeks of pregnancy. The objective of this study was to identify potential immune-related biomarkers and molecular subtypes for the treatment of PE.

**Methods:**

Three datasets of GSE10588, GSE25906 and GSE48424 were downloaded from the Gene Expression Omnibus (GEO) database. The names of immune-related genes were retrieved from the ImmPort immune database. To screen the differentially expressed immune-related genes, the “limma” R package was used. An analysis of logistic regression was used to identify the key genes and a nomogram was constructed using these key genes. These key gene expression profiles were further validated using qRT-PCR. In addition, the landscape of immune cell infiltration was investigated using the CIBERSORTX software. The potential molecular subtypes of PE were also investigated using the “ConsensusClusterPlus” R package.

**Results:**

The 103 immune-related genes differentially expressed were identified, including 47 up-regulated genes and 56 down-regulated genes. Univariate and multivariate logistic regression analysis was used to screen five key genes, including *CCL24, ENG, LCP2, GNAI1* and *FLT3*. The key genes were strongly associated with immune cell infiltration. Two molecular subtypes (C1 and C2) were identified. Both exhibited distinct levels of immune cell infiltration and gene expression.

**Conclusion:**

This study identified five key genes, as well as immune-related subtypes, that could provide potential therapeutic targets and aid in the design of more precise PE immunotherapy.

**Supplementary Information:**

The online version contains supplementary material available at 10.1186/s41065-025-00458-9.

## Background

PE is characterized by high blood pressure and massive proteinuria after 20 weeks of pregnancy [1], potentially leading to maternal mortality and adverse outcomes for newborns [2]. There are 50 000 to 60 000 PE-related maternal deaths worldwide annually [3]. PE is highly correlated with the incidence rate, mortality rate, maternal organ dysfunction [4], or fetal growth restriction [5], and the increased risk of cardiovascular disease [6], leading to a significant economic and psychological costs for families and society.

Currently, the etiology and pathogenesis of PE are not fully understood. Immune changes are widely regarded as the primary cause of PE [7]. The pathological basis of disease occurrence is shallow placental implantation. Villous trophoblasts and the maternal immune system adapt mutually to form placentas. When specific immune factors cause maternal and infant immune intolerance, placental formation is poor, resulting in disease [8]. Pregnancy can be regarded as a semi-allogeneic transplantation phenomenon. The foundation for a successful pregnancy is the establishment and maintenance of the immune balance between mother and fetus. The role of immune system imbalance in its pathogenesis has attracted a lot of attention [9]. The maternal–fetal interface contains a variety of cells such as decidual stromal cells, immune cells, trophoblast cells, and others [10]. Through research, Jonsson et al. supported the hypothesis of increased inflammatory response in PE [11]. PE immunopathological has been explored, for example: There is an association between PE and acute atherosclerosis, characterized by lipid-laden foam cells within the intima, which lead to poor placental development [12]. Several studies have found that impaired dNK-trophoblast interaction may result in poor placentation, resulting in PE and other pregnancy pathological conditions [13]. Consequently, understanding the cellular immune changes and interactions may help in the development of immunotherapy strategies for this syndrome.

The advancements in high-throughput sequencing and bioinformatics have provided valuable information for determining the genetic dynamics of specific diseases. Tea kaartokallio et al., sequenced the placental transcriptomes of 9 PE patients and 9 healthy pregnant women, and identified 53 differentially expressed gene [14]. Liu et al. identified 17 differentially expressed hub genes and can be used as potential biomarkers for diagnosis of PE [15]. Li et al. elaborated on PE-related modules and hub genes using weighted gene co-expression network analysis and elucidated that LDHA might be a therapeutic target in PE [16]. Gao et al. revealed CADM3 might be served as a potential marker for the diagnosis of PE by integrating multiple cohorts [17]. Sheng et al. found that TYROBP, PLEK, LCP2, HCK, and ITGAM are strongly associated with preeclampsia, suggesting they could serve as biomarkers for diagnosis and help improve understanding of its development [18]. Despite extensive research on PE, there are still many new candidate mechanisms yet to be explored or unknown in its pathogenesis, further clarification and research are needed on the pathogenesis of this multisystem disease.

In this study, we integrated three microarray datasets from the GEO database to identify five immune-related genes that could be used as a therapeutic target for PE. Moreover, we identified three immune-related molecular subtypes, providing a basis for understanding the molecular mechanism and treatment of PE.

## Materials and methods

### Selection of the GEO dataset and data processing

The gene expression microarray datasets of GSE10588, GSE25906 and GSE48424 were obtained from the GEO database (http://www.ncbi.nlm.nih.gov/geo/). GSE10588, based on the GPL2968 platform, included 26 control samples and 17 PE samples. GSE25906, based on the GPL6102 platform, contained 37 control samples and 23 PE samples. GSE48424, based on the GPL6480 platform and comprised of 18 control and PE samples. Among these cohorts, the samples from GSE10588 and GSE25906 were derived from placenta tissue, while samples of GSE48424 were collected from blood tissue. Considering the chip data measured by different GPL versions, the “sva” R package (version 3.56.0) was used to remove the batch effects, and then PCA analysis was used to evaluate the distribution of samples before and after correction [19].

### Differential expression analysis

The immune-related gene list was retrieved using the IMMPORT immune database (https://www.immport.org/). The “limma” R package (version 3.64.0) was applied to identify the differential expression genes in the merged cohort [20]. Up-regulated genes were defined as those with a log2 Fold Change > 0 and corrected *P*-values < 0.05, whereas down-regulated genes were those with a log2 Fold Change < 0 and corrected *P*-values < 0.05.

### Functional and pathway enrichment analyses of DEGs

To investigate the biological behaviors of these DEGs, Gene Ontology (GO) and Kyoto Encyclopedia of Genes and Genomes (KEGG) enrichment analysis were performed by using the “clusterProfiler” R package (version 4.16.0) [21]. The significant functions or pathways were screened based on the criterion: adjusted *p* value < 0.05.

### PPI network construction and module analysis

STRING (version 10.5) (http://string-db.org/) online tools was used to build PPI networks [22], and the interactions were filtered with a medium confidence level more than 0.7. Subsequently, all the interactions were visualized by using cytoscape software (http://www.cytoscape.org/, version 3.6.1) [23]. MCODE module from cytoscape software was applied to investigate key interaction modules with degree cutoff = 2 and node score cutoff = 0.2 [24].

### Logistic regression analysis

After selection of the key modules, we then performed univariate and multivariate logistic regression analyses to screen genes based on them. The genes with a *p*-value < 0.05 were considered significant. To evaluate the diagnostic power of these key genes, we also conducted receiver operating characteristic curve (ROC) analysis between control and PE samples through “pROC” R package (https://cran.r-project.org/web/packages/pROC/index.html, version 1.8.15) [25].

### Construction and evaluation of nomogram prediction model

The nomogram was constructed using the “rms” (https://cran.r-project.org/web/packages/rms/index.html, version 8.0) package based on the key genes from the logistic regression analysis result. The calibration curve was used to evaluate the accuracy of the nomogram. The clinical benefit of the nomogram was calculated using decision curve analysis (DCA).

### Functional identification of hub genes

To explore the potential involved pathways of the genes, we categorized patients into high and low expression group based on their median expression level. Subsequently, the potential pathways were identified using gene set enrichment analysis (GSEA) through “GSVA” R package (version 2.2.0), with a normal *P* -value < 0.05 considered statistically significant [26].

### Correlation between hub genes and immunity

To further evaluate the relationship between key genes and immune cells, we uploaded the gene expression profile of all samples to the CIBERSORTX website (https://cibersortx.stanford.edu/) [27]. The difference in immune cell infiltration between control samples and PE samples were then compared. Furthermore, Pearson correlation analysis was used to estimate the relationship between genes and immune cells.

### Consensus clustering analysis

Based on the differential expression of immune-related genes, the “ConsensusClusterPlus” R software (version 1.72.0) was used to investigate the potential molecular subtypes of PE [28]. The following parameters were used: reps = 50, pItem = 0.8, clusterAlg = “km” (k-means clustering) and distance = euclidean (Euclidean distance metric).

### Placental tissue collection

We collected 48 placenta specimens between August 2021 and March 2022, with 24 specimens from patients with PE and 24 from healthy pregnant women at the Department of Gynecology and Obstetrics, The First Affiliated Hospital of USTC (Hefei, China). Diagnosed criteria of PE is based on the international federation of gynecology and obstetrics (FIGO) initiative on pre-eclampsia [29].

Within 30 min of delivery, placental tissue sections of 1 cm^3^ that does not contain the decidua basalis were randomly taken from the placenta near the root of the umbilical cord under strict aseptic conditions. Embolic and calcified sites were avoided. The sample was stored at −80˚C for RT-qPCR analysis.

### PCR gene expression changes in PE

For evaluation of the key gene expression in these two groups, RNA was extracted and reverse transcribed into cDNA for 30 min. qPCR was performed on a Real-Time PCR Detection system. The 2-ΔΔCq method was used to quantify the relative mRNA levels [30]. Critical Technologies: GAPDH, sense 5′- GAAGGTGAAGGTCGGAGTCAA-3′; antisense 5′- CTGGAAGATGGTGATGGGATTT-3′. β-actin, sense 5′-CCCTGGAGAAGAGCTACGAG-3’, antisense 3′-GGAAGGAAGGCTGGAAGAGT-5’. LCP2 sense, 5′- AGGAGCATCTTCACACGCAA-3′; antisense 3′-CCATTGTCCTCTTCGTGGCT-5′. Eng sense: 5′- TGCACTTGGCCTACAATTCCA-3′, antisense 3′-AGCTGCCCACTCAAGGATCT-5′. GNAI1 sense: 5′- GATGATGCACGCCAACTCTT-3′, antisense 3′-CTCCAGCAAGTTCTGCAGTC-5′. CCL24 sense: 5′- AAGGACCCGAGCTATTTATC-3′,antisense 3′-CATGTCTCAGAGAGCAGAAG-5 ‘. FLT3 sense:5’-CGCTGCTCGTTGTTTT-3’, antisense 3’-GATGACTTCCCCACTGATG-5.

### Statistical analysis

All analyses were conducted in the R (version 4.2.2) and GraghPad Prism software (version 7.0). The Wilcoxon rank-sum test or Student's t-test was used to estimate the difference between two groups. Kruskal–Wallis test was appied to evaluate difference of three groups. Chi-square test or fisher test was used to calculate differences in categorical groups. For all analyses, *P*< 0.05 was considered significant.

## Results

### Identification of DEGs between PE and control samples

**Fig. 1:**
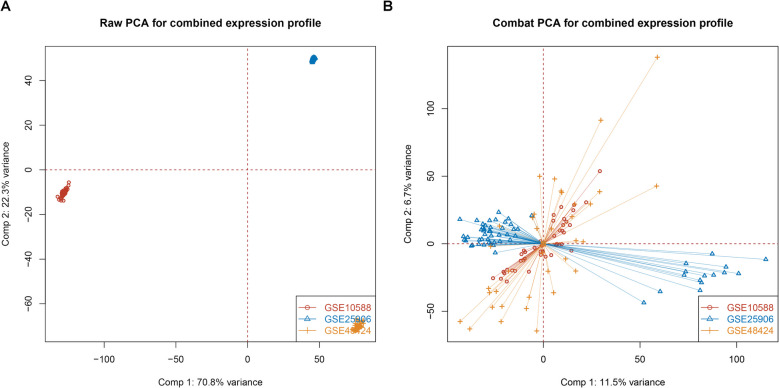
Evaluation of the batch effect before (**A**) and after (**B**) merging through the principal component analysis

**Fig. 2:**
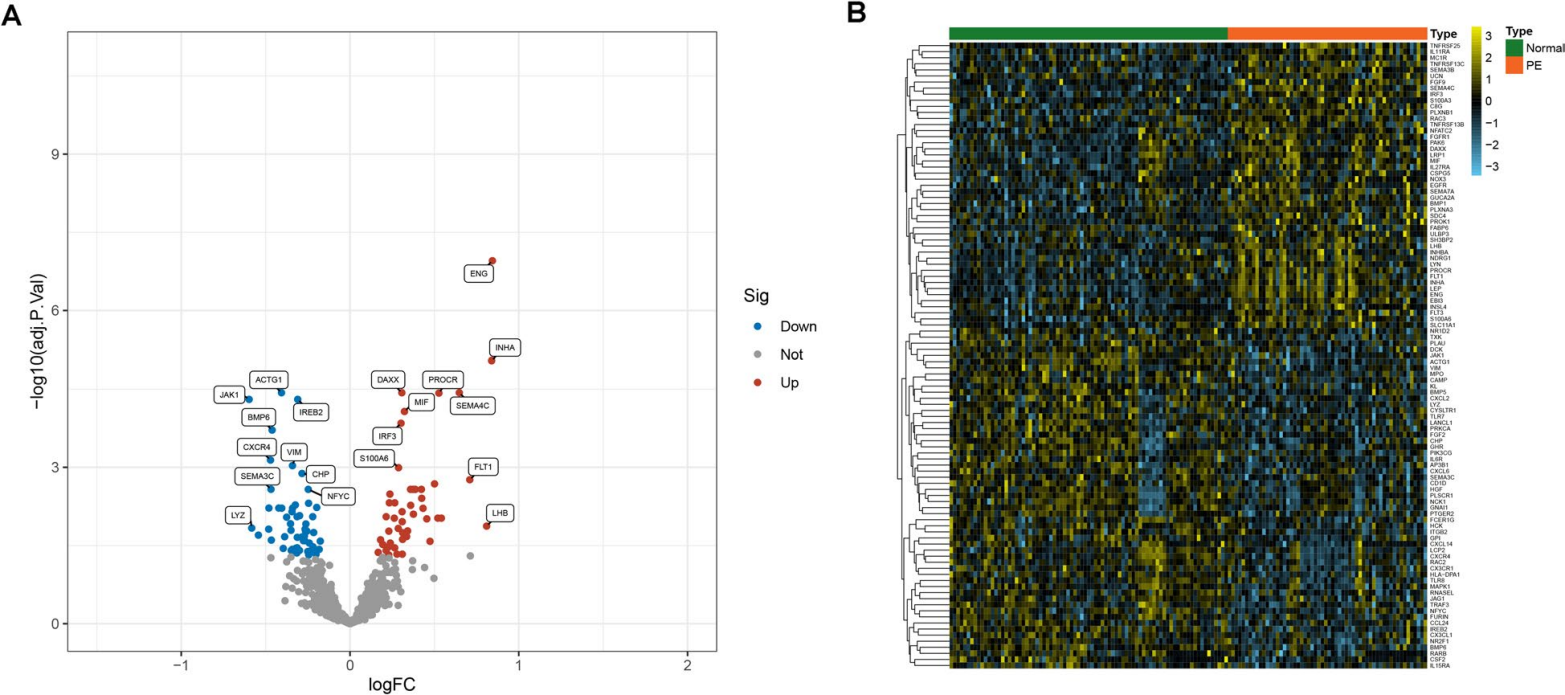
Identification of differentially expressed genes in the merged dataset. **A** Volcano plot of the genes, the green dots represent the down-regulated genes, red dots represent the up-regulated genes, while the black dots showed genes with no significance. **B** A heatmap plot of the differentially expressed genes

The GSE10588, GSE25906 and GSE48424 were three datasets that included 81 normal samples and 58 disease samples in total. Using the chip data measured by various GPL versions, we removed the batch effect between the datasets using the “sva” R package. PCA analysis was used to compare sample distribution before and after correction. The samples were mixed as shown in Fig. 1A and B, revealing that the batch effects had been removed. We then used the limma R package to compare the gene expression between normal and PE samples. As a result, 103 immune-related DEGs were screened, with 47 up-regulated genes and 56 down-regulated genes. Figure 2A and B show the distribution and expression of DEGs, respectively.

## Function enrichment analysis of the DEGs

**Fig. 3:**
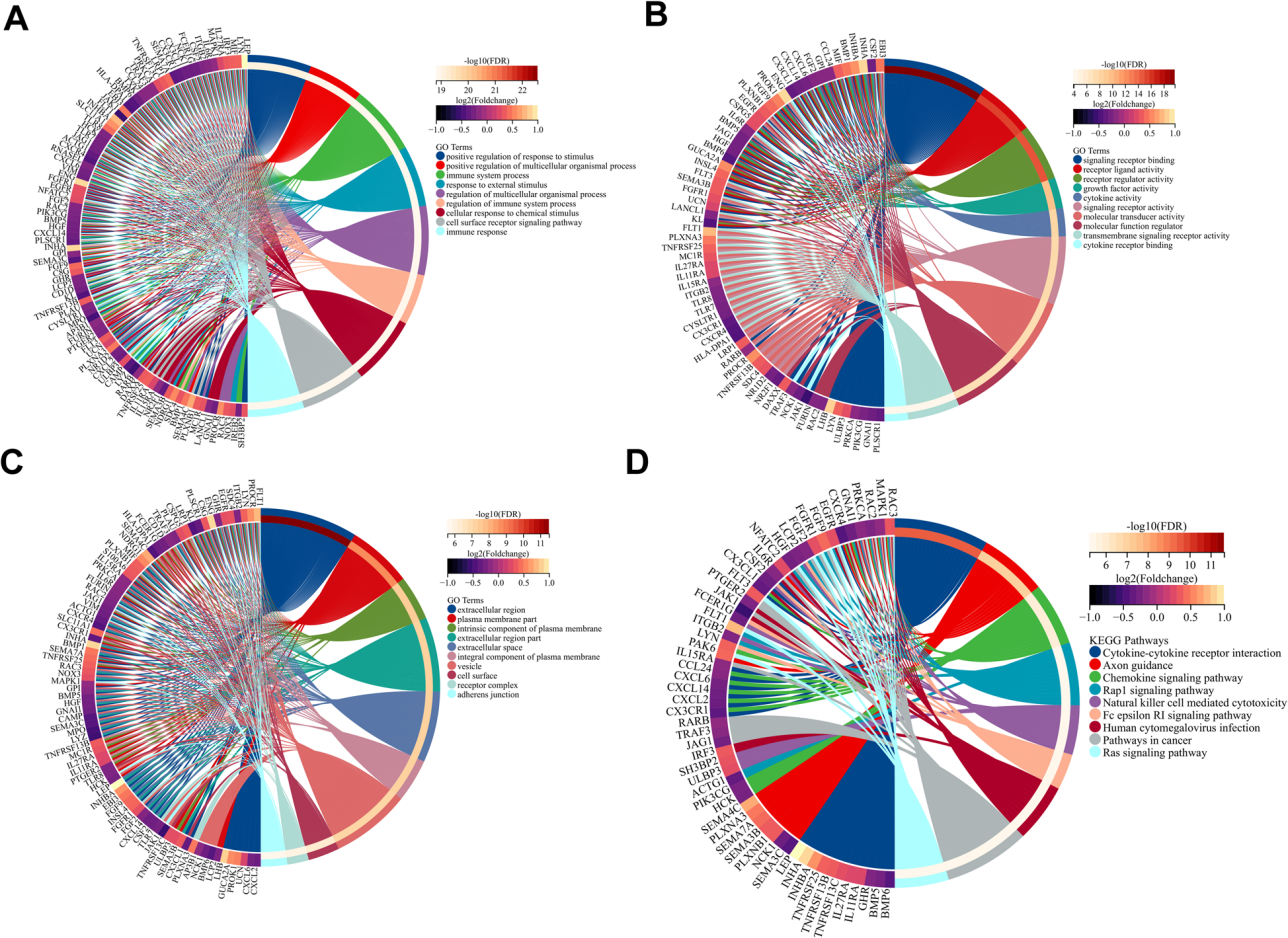
Gene ontology and KEGG enrichment analysis results of the DEGs. **A**-**C** GO enrichment analysis including BP, MF, CC, and (**D**) KEGG pathway enrichment analysis

Three components were identified based on the GO enrichment analysis results: Biological Process (BP), Molecular Function (MF), and Cellular Component (CC). Positive regulation of response to stimulus, immune system process, immune system regulation process and response to external stimulus were all enriched in BP (Fig. 3A). Signaling receptor binding, receptor-ligated activity, receptor regulator activity, growth factor activity and cytokine activity were all components of MF (Fig. 3B). The CC was enriched in the extracellular region, plasma membrane part, an intrinsic component of the plasma membrane and extracellular region part (Fig. 3C). The KEGG pathway enrichment included cytokine-cytokine receptor interaction, chemokine signaling pathway, Rap1 signaling pathway and Natural killer cell-mediated cytotoxicity (Fig. 3D). All these findings suggested that these DEGs could play a critical role in the development of PE.

### PPI networks and key genes identification

**Fig. 4:**
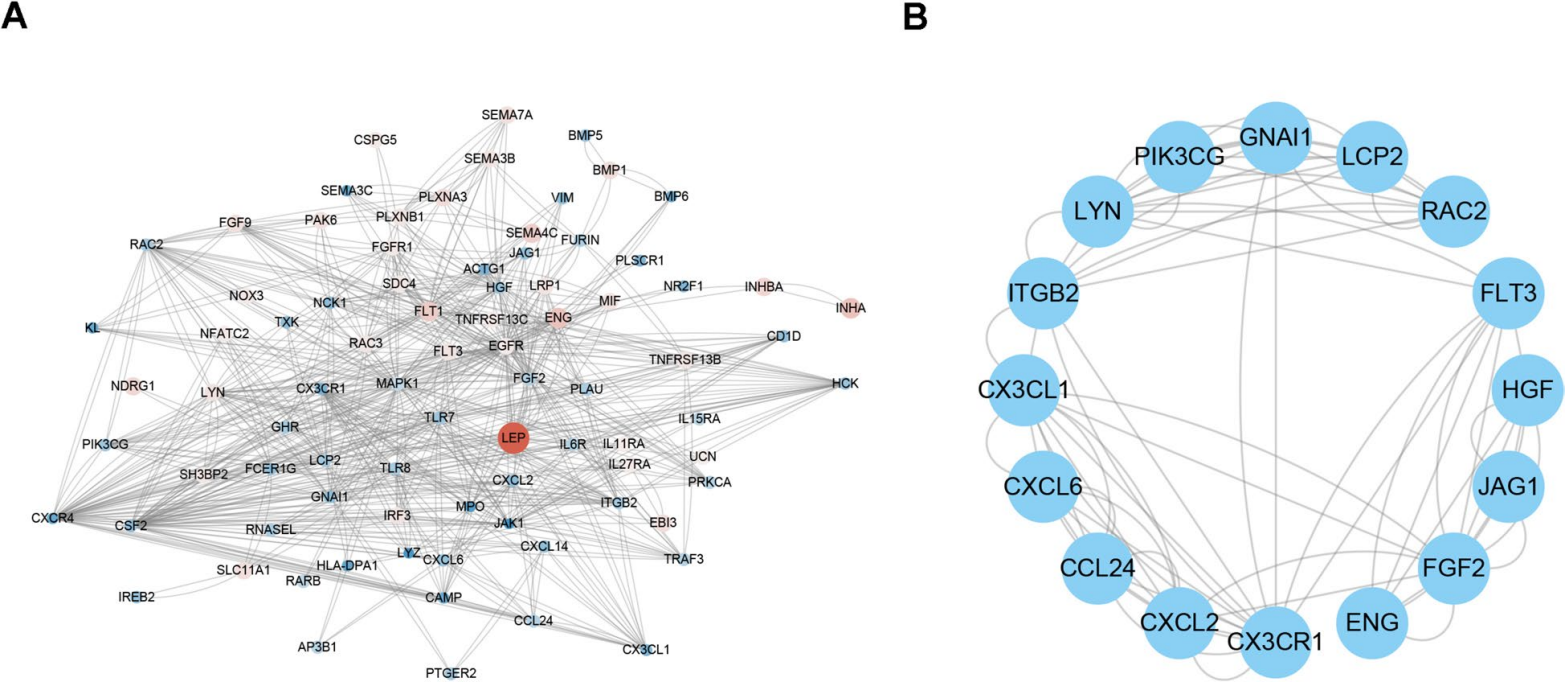
The protein interaction network diagram. **A** The protein interaction diagram of all differential genes. **B** The core gene interaction diagram screened by the MCODE algorithm

**Fig. 5:**
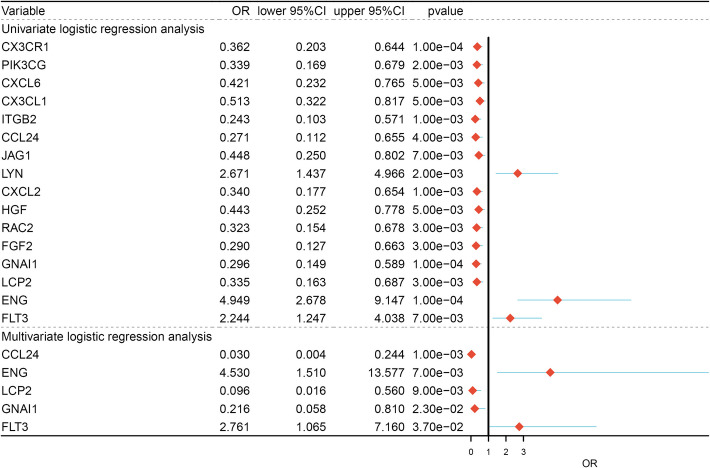
Univariate and multivariate logistic regression analysis forest plot

To investigate the interactions between the immune-related DEGs of PE, a protein–protein interaction network (PPI) was constructed using the STRING database (https://string-db.org/). A total of 694 protein interaction pairs were identified and then visualized using cytoscape software (Fig. 4A). The MCODE module was then used to screen the hub network from the 694 protein interaction pairs (Fig. 4B). Finally, we performed univariate and multivariate logistic regression analysis to identify the key genes associated with PE based on the hub network, and five key genes CCL24, ENG, LCP2, GNAI1 and FLT3 were identified (Fig. 5). Among them, ENG and FLT3 were up-regulated in the PE tissue, while CCL24, LCP2 and GNAI1 were highly expressed in normal tissue.

### The diagnostic value of key genes and nomogram construction

**Fig. 6:**
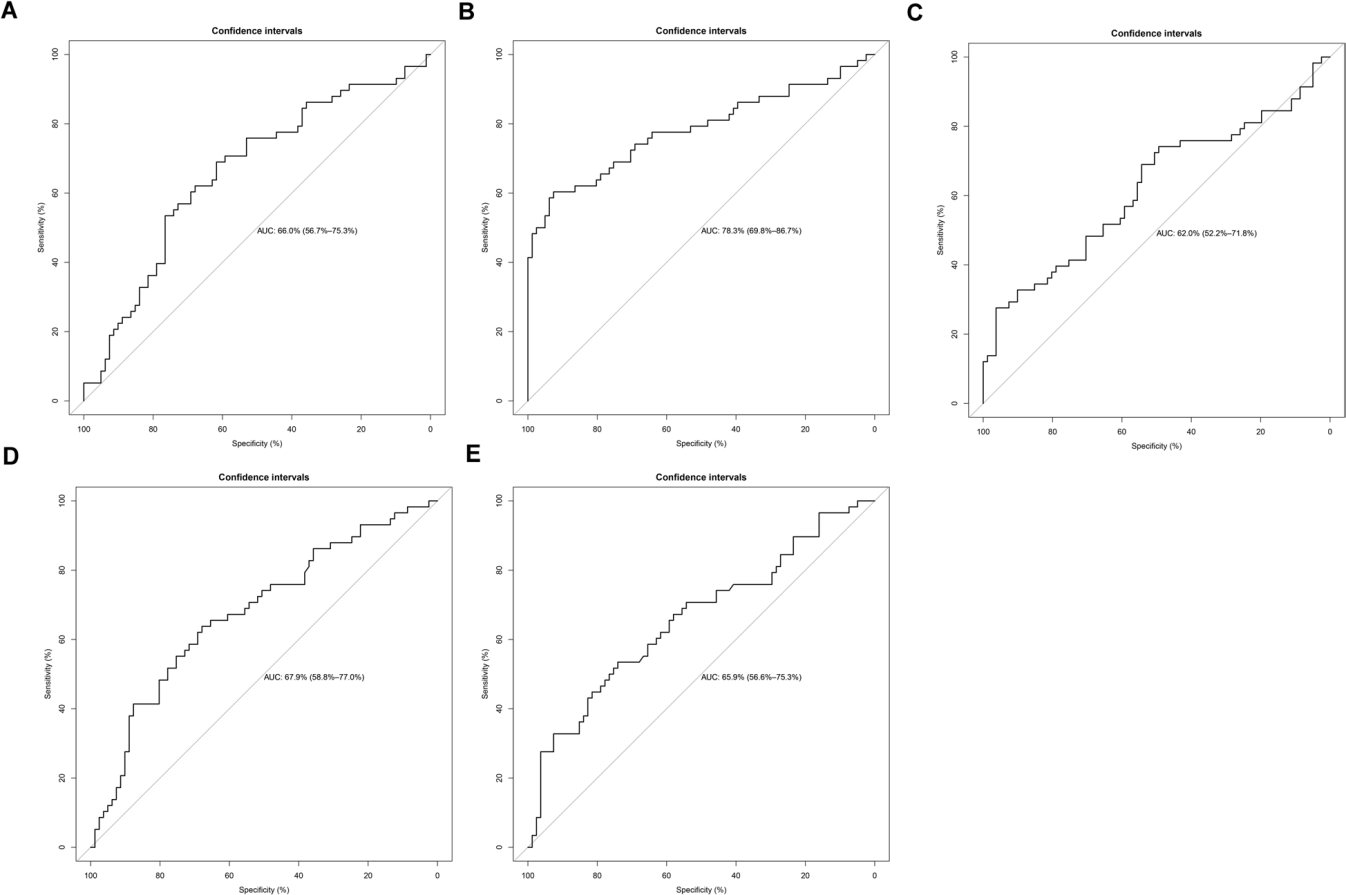
Nomogram was constructed to estimate the probability of PE using the 5 key genes. **B** Calibration curve for estimating the agreement between our predicted values and reality. **C** Decision tree curve for estimating whether the model-based decision would benefit the patient

**Fig. 7:**
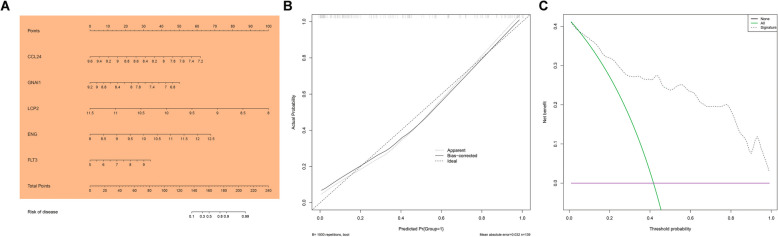
Using ROC curve was used to evaluate the diagnostic value of 5 key genes. **A** CCL24, (**B**) ENG, (**C**) FLT3, (**D**) GNAI1,(**E**) LCP2

The diagnostic value of the five key genes was assessed using ROC analysis. The AUC value for CCL24, ENG, FLT3, GNAI1 and LCP2 were 0.660, 0.783, 0.620, 0.679 and 0.659, respectively, as shown in Fig. 6, indicating a good performance between PE and normal samples and can be used as a potential biomarker for the PE diagnosis. Additionally, we also created a nomogram for predicting PE based on the expression level of five key genes (Fig. 7A). The prediction probability of nomogram and the actual probability were well-matched based on the calibration curve (Fig. 7B). The DCA result revealed that the nomogram had some clinical benefits for predicting PE (Fig. 7C).

### The molecular function of the five key genes

**Fig. 8:**
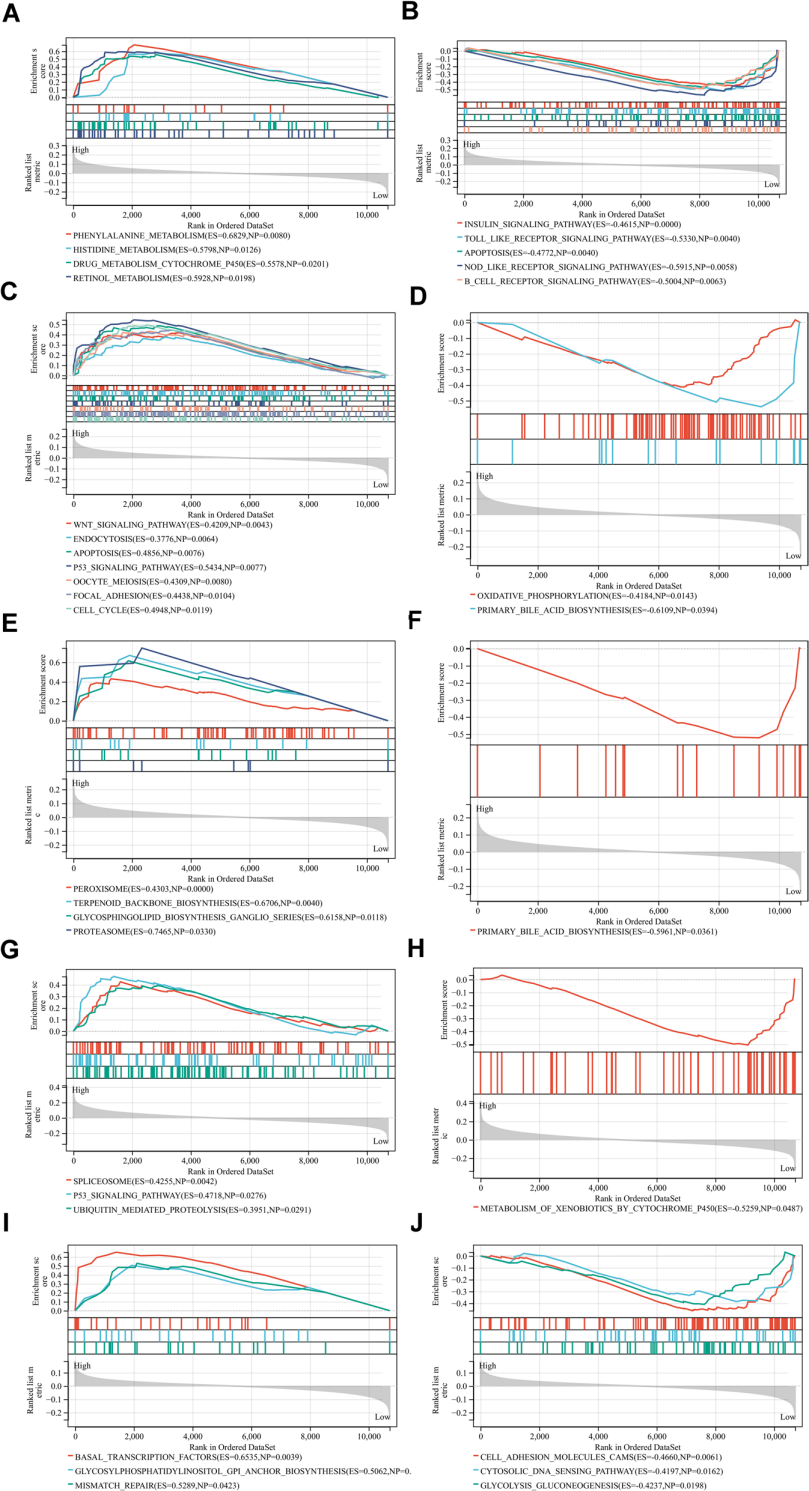
Functional enrichment analysis of 5 key genes, (**A**-**B**) CCL24, (**C**-**D**) ENG, (**E**–**F**) FLT3, (**G**-**H**) GNAI1, (**I**-**J**) LCP2

The molecular function of the five key genes was evaluated by the Gene Set Enrichment Analysis (GSEA). All genes were categorized into high and low-expression groups, according to the median gene expression. Phenylalanine metabolism, histidine metabolism and retinol metabolism were enriched in the high expression group of CCL24 (Fig. 8A). On the other hand, apoptosis B cell receptor signaling pathway and Toll-like receptor signaling pathway were enriched in the low-expression group of CCL24 (Fig. 8B). The WNT signaling pathway, p53 signaling pathway, cell cycle and focal adhesion were significantly enriched in the high-expression group of ENG, whereas oxidative phosphorylation was significantly enriched in the low-expression group (Fig. 8C-D). Peroxisome, terpenoid backbone biosynthesis and proteasome were enriched in the FLT3 high-expression group, whereas primary bile acid biosynthesis was enriched in the FLT3 low-expression group (Fig. 8E-F). The P53 signaling pathway and spliceosome were significantly enriched in the GNAI1 high expression group (Fig. 8G), whereas the metabolism of xenobiotics by cytochrome P450 was enriched in the GNAI1 low expression group (Fig. 8H). Finally, mismatch repair and basal transcription factors pathways were identified in the LCP2 high-expression group (Fig. 8I), while cell adhesion molecules cams and cytosolic DNA sensing pathways were enriched in the low-expression group (Fig. 8J).

### The immune landscape of PE

**Fig. 9:**
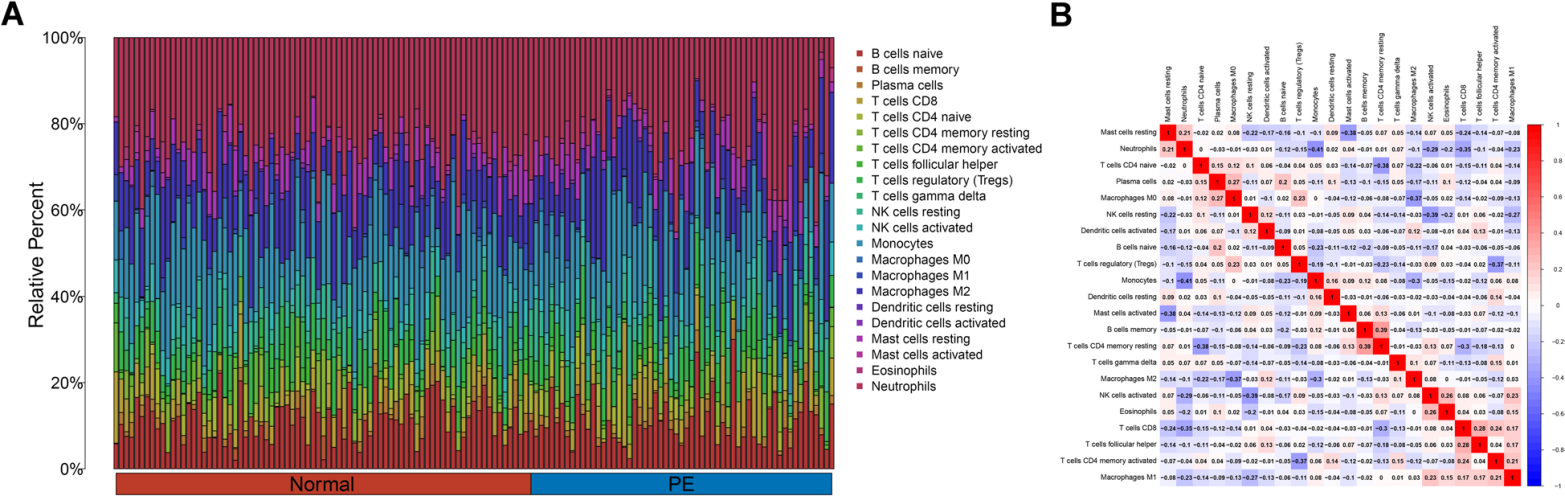
Differences of 22 types of immune cells in normal and PE tissue. A comparison of the 22 immune cells (**A**) and correlation of immune cells (**B**) between normal and PE samples

**Fig. 10:**
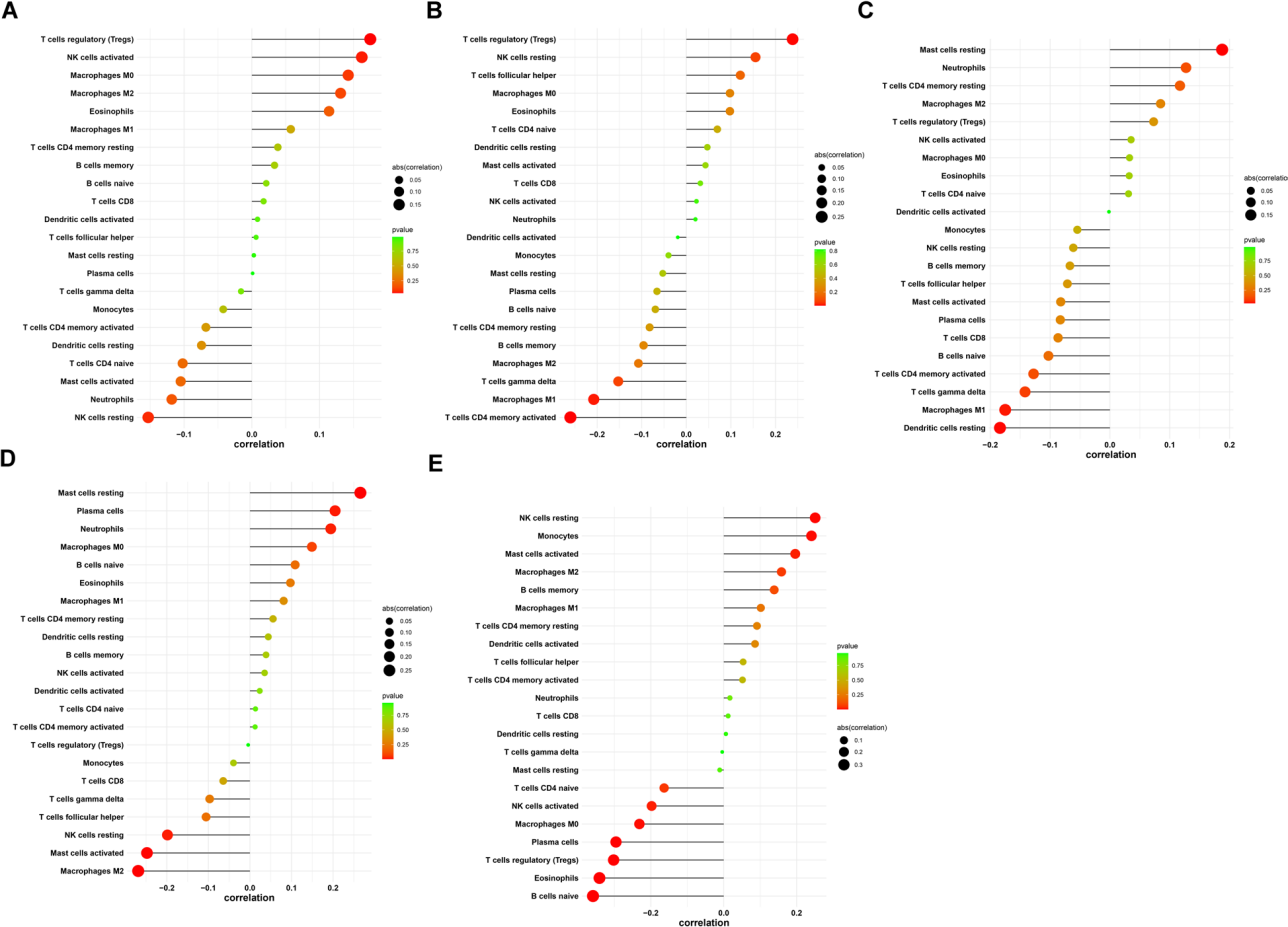
The correlation of 5 hub genes with immune cells. **A **CCL24, (**B**) ENG, (**C**) FLT3, (**D**) GNAI1, (**E**) LCP2

The CIBERSORTX software was used to estimate the 22 immune cells of each sample in PE (Fig. 9A). The neutrophils had the strongest negative correlation with monocyte (cor = −0.41), while T follicular helper cells had positive correlation with CD8 T cells(cor = 0.28) (Fig. 9B). Moreover, we discovered that there is significance in M1 macrophages and resting mast cells (Figure S1). We further assessed the relationship between 22 immune cells and five key genes using correlation analysis. Regulatory T cells (Tregs) were strongly positively correlated with CCL24 (Fig. 10A) and ENG (Fig. 10B) while resting mast cells were correlated with FLT3 (Fig. 10C) and GNAI1 (Fig. 10D), while LCP2 was strongly positively correlated with resting NK cells (Fig. 10E). Furthermore, CCL24, ENG, FLT3, GNAI1 and LCP2 were most negatively related to the resting NK cells, activated CD4 memory T cells, resting dendritic cells, M2 macrophages and naive B cells, respectively.

### Identification of molecular subtypes of PE

**Fig. 11:**
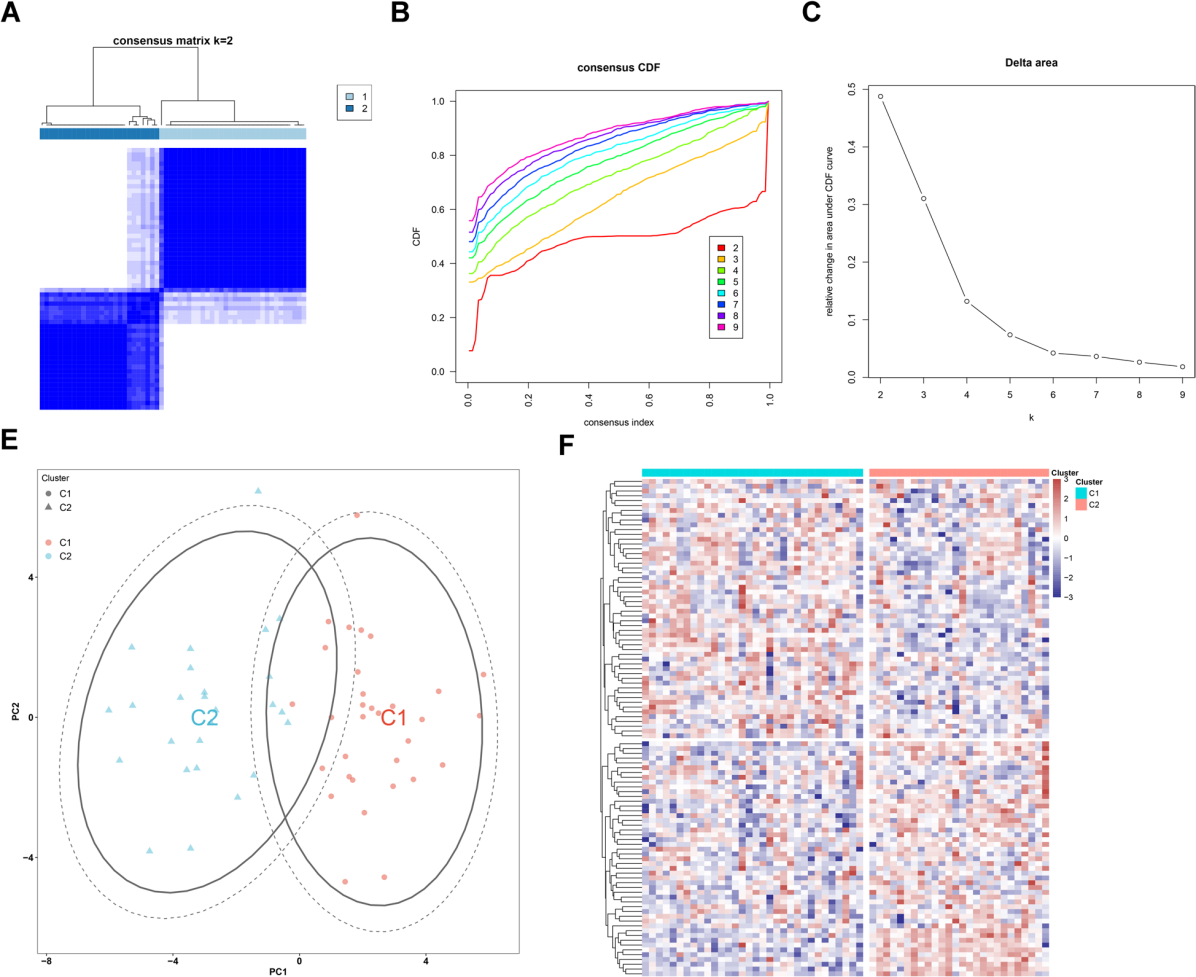
Identification of potential subtypes of PE based on immune differential genes. **A**-**C** Cumulative distribution function and heatmap to indicate that the optimal number of subtypes is 2. **E** PCA analysis of the differences between the two subtypes. **F** The number of genes in the subtypes Express situation

**Fig. 12:**
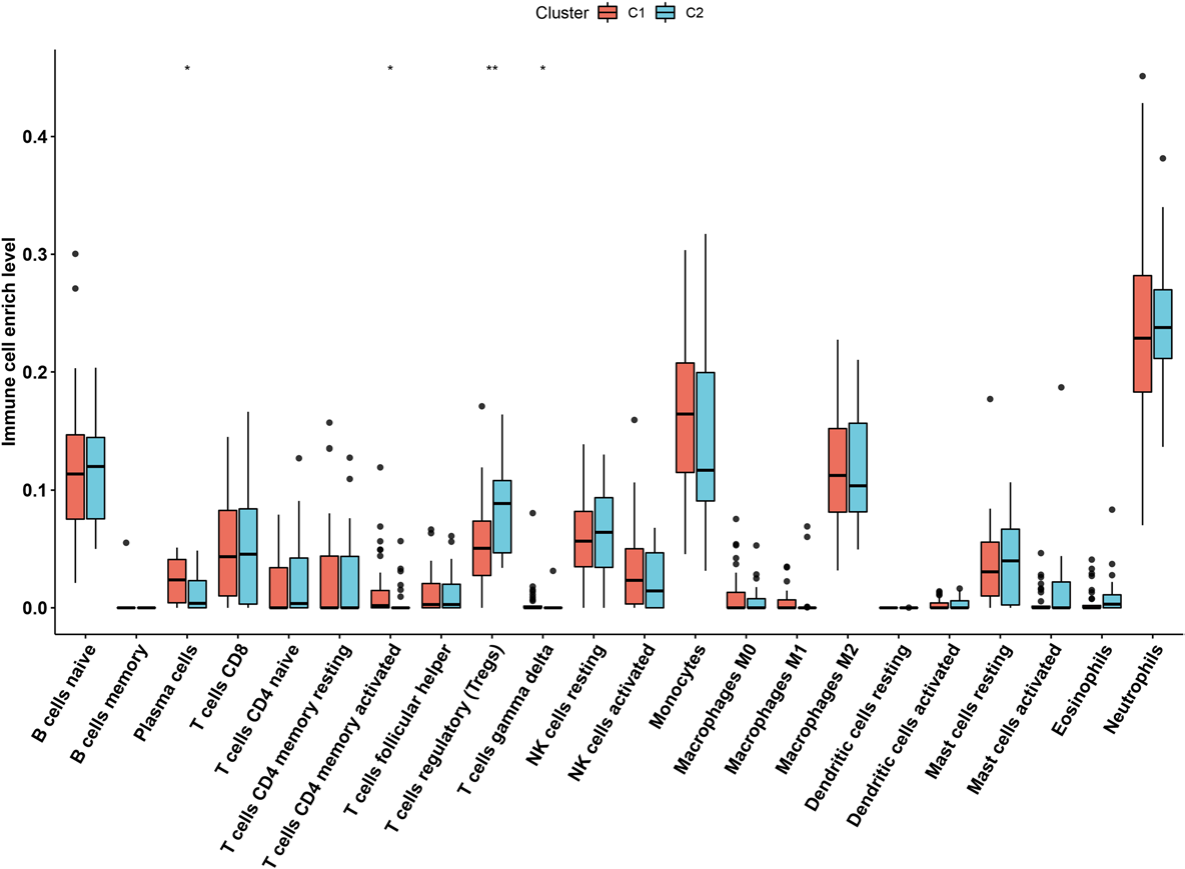
Immune cell differences among subtypes

We performed a sample clustering analysis based on the differential expression of immune-related genes to investigate the immune-related molecular subtype of PE (Fig. 11A-C). We finally choose K = 2 as the optimal cluster based on the cumulative distribution function (CDF) and delta value after a comprehensive consideration. Moreover, to validate the cluster assignments, we also performed PCA analysis to decrease the dimension of features and found the subtypes designations were largely consistent with the distribution of PCA result (Fig. 11D). We found the expression level of the differentially expressed immune-related genes were showed specific expression in subtypes (Fig. 11E). We further evaluated the immune cell infiltration level of the two subtypes. As showed in Fig. 12, we found that gamma delta T cells, plasma cells and activated CD4 memory T cells were highly expressed in subtype C1, while Regulatory T cells (Tregs) showed a high infiltration level in C2.

## RNA extraction and quantitative real-time PCR

**Fig. 13:**
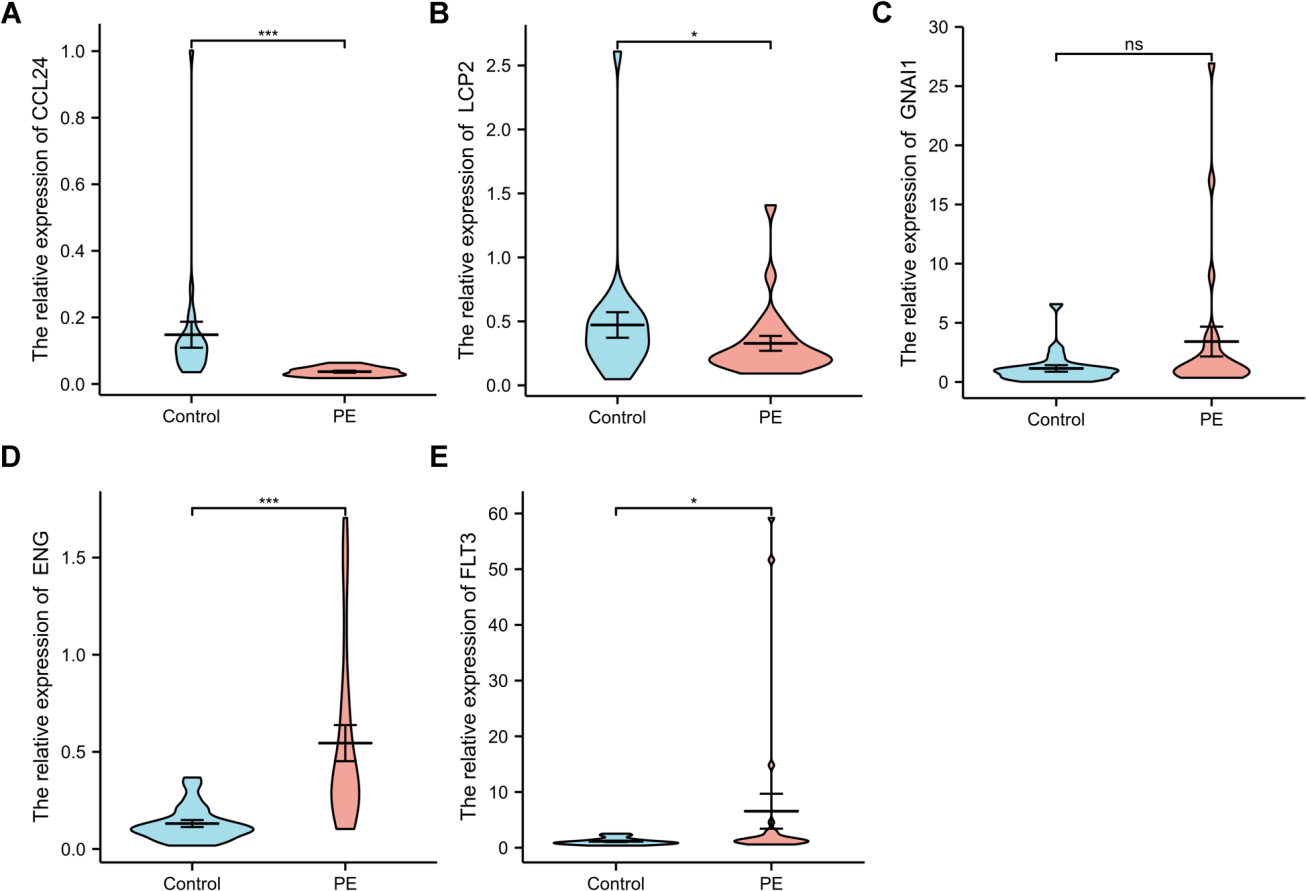
The relative expression of 5 key genes in placenta samples from patients between PE and control groups. * *P*< 0.05; **P< 0.01. **A** CCL24, (**B**) LCP2, (**C**) GNAI1, (**D**) ENG, (**E**) FLT3

To validate the expression level of the five key genes, we compared the PE and normal tissue through the qRT-PCR experiment. As shown in Fig. 13, the expression level of CCL24 and LCP2 in normal tissue was significantly higher than those in the placenta of patients with PE. On the other hand, ENG and FLT3 presented a high expression level in PE, consistent with our previous findings. However, there was no difference in GNAI1 expression levels between PE tissue and normal placenta.

## Discussion

PE is a vascular system disorder that develops during pregnancy in women who had normal blood pressure before the second half of pregnancy, along with hypertension and proteinuria [31]. Approximately 3% of pregnancies are affected by PE, making it the primary cause of maternal and fetal mortality globally [32]. The etiology of PE is currently unknown, but emerging evidence indicates that immune responses play a crucial role in its development. Immune system activation is the most common immunological finding in congenital eclampsia [33]. Therefore, it is critical to uncover the role of immune-related genes during the development of PE.

In this study, we analyzed microarray datasets to pinpoint hub genes as potential therapeutic biomarkers for PE. A total of 103 dysregulated DEGs (47 up-regulated and 56 down-regulated) linked to PE were identified through the analysis of three datasets. Logistic regression analysis and PPI network analysis identified five key genes for PE: CCL24, ENG, LCP2, GNAI1, and FLT3. These genes showed good diagnostic value and can serve as potential biomarkers for PE diagnosis and therapy. ENG is a commonly studied serum marker for PE. The expression of ENG in placental samples from pregnant women with PE is notably higher in both early and late pregnancy compared to control [34], consistent with our findings. Recent studies found that ENG has been involved in the regulation of placental trophoblast cell differentiation, as well as participating in the maintenance of vascular tension [35]. CCL24 (chemokine C–C motif ligand 24) can promote eosinophil migration to the lungs by up-regulating the adhesion of endothelial cells [36]. Additionally, CCL24 has been linked to inflammation and fibrosis in the lungs and skin [37]. There is growing evidence that GNAI1 plays a role in human platelet aggregation [38], signal transduction of rat liver trypsin receptor, paclitaxel resistance of human ovarian cancer cells [39], hypotonia and epilepsy [40]. The LCP2, also known as SLP-76, is an adaptive protein-coding gene, which is essential for normal T cell development, activation and serine phosphorylation activation [41]. In addition to T cells, LCP2 also play an important role in NK cell-mediated self deletion target recognition [42], as well as actively regulates antigen-induced mast cell activation by BCR recruitment [43]. LCP2 deficiency can result in excessive production of pro-inflammatory cytokines [44]. The FLT3 is expressed by a variety of cells, including hematopoietic cells and bone marrow stromal cells [45]. Moreover, FLT3 plays an important role in immune system activation by stimulating the production of dendritic cells and natural killer cells [45].

PE patients exhibit placental dysfunction and inadequate uterine spiral artery transformation, potentially compromising mothers' immune tolerances due to this defect in the placenta [46]. In cases where the maternal immune system is changed due to potential autoimmune disorders, it might interfere with the immune adaptation of the placenta, thus raising the risk of PE [47]. The level of M1 macrophages in our study showed a significant difference between PE and normal controls, suggesting that M1 macrophages play an important role in PE. Studies have revealed that an imbalance in the M1/M2 macrophage ratio is linked to pregnancy-related diseases [48]. In patients with PE, the functional maturation of macrophages is impaired, and there is a pro-inflammatory imbalance dominated by phenotype M1 [49]. The abnormal phenotype and function of placental decidual macrophages will affect the imbalance of the microenvironment at the maternal–fetal interface as well as the destruction of immune tolerance, ultimately leading to the development of PE [50]. Moreover, there was a significant difference between PE and normal control in the resting mast cell. Histamine and prostaglandins released by activated mast cells may be linked to vasospasm in PE [51]. Hence, our immune-related classification may help to show the immune landscape of PE, which may aid in early diagnosis and PE treatment.

Despite five potential biomarkers and two molecular subtypes associated with PE were identified in our study, several limitations should be elucidated. Firstly, the sample size in this study is relative low and should be enlarged in future studies. Secondly, the molecular mechanism of key genes should be validated through in-vivo and -vitro experiment.

## Conclusion

This study identified five key genes (CCL24, ENG, LCP2, GNAI1 and FLT3) that can serve as effective therapeutic biomarkers for PE. Furthermore, we identified two molecular immune-related subtypes associated with PE, which might contribute to the prevention and personalized treatment of PE.

### Limitations

Despite identifying five potential biomarkers and two molecular subtypes associated with PE, several limitations should be elucidated. Firstly, the sample size in this study is low and should be enlarged in future studies. Secondly, the gene function should be validated through extensive trials.

## Supplementary Information

Supplementary Material 1. Figure S1. Immune cell infiltration between PE and control groups.

## Data Availability

The datasets used in this study are available in the GEO database repository, (http://www.ncbi.nlm.nih.gov/geo/).
