# Supplementary figures and images for: Identification of immune-related genes and molecular subtypes associated with preeclampsia via bioinformatics analysis and experimental validation
Source: Hereditas. 2025 May 29;162:89. doi: 10.1186/s41065-025-00458-9 (PMC12123883; doi:10.1186/s41065-025-00458-9)

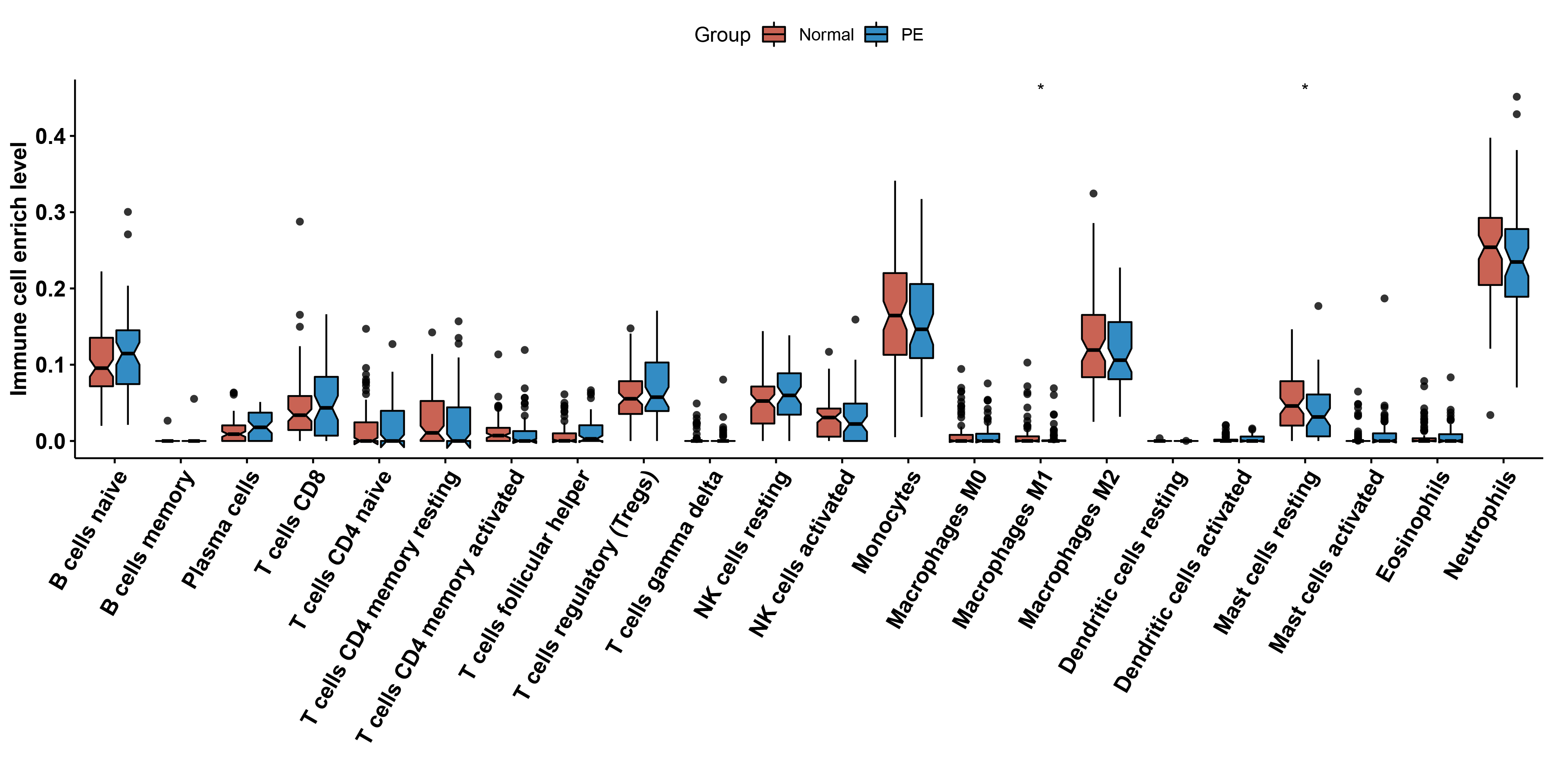

Supplement: Supplementary file 1 — Supplementary Material 1. Figure S1. Immune cell infiltration between PE and control groups. [file 41065_2025_458_MOESM1_ESM.jpg]
